# How teacher–student collaborative assessment relates to L2 writing self-efficacy: insights from Chinese EFL learners

**DOI:** 10.3389/fpsyg.2026.1759376

**Published:** 2026-05-13

**Authors:** Nan Huang, Chenming Lyu

**Affiliations:** School of Foreign Languages, Ludong University, Yantai, China

**Keywords:** EFL learners, L2 writing, secondary school writing, student subgroup patterns, teacher–student collaborative assessment, writing self-efficacy

## Abstract

**Objective:**

Teacher–student collaborative assessment (TSCA) has attracted increasing attention as a participatory assessment approach in L2 writing, yet its relationship with writing self-efficacy remains underexplored, particularly in secondary-level EFL contexts. This study aimed to examine whether TSCA was associated with changes in students’ L2 writing self-efficacy and whether different patterns emerged across gender and internally defined pretest performance subgroups.

**Methods:**

A quasi-experimental pretest–posttest design was implemented with two intact Grade 10 classes in a Chinese senior high school. One class received TSCA-based writing instruction, whereas the other followed a traditional teacher-centered assessment approach. Writing self-efficacy was measured using an adapted questionnaire, and supplementary qualitative data were collected through semi-structured interviews. Quantitative data were analyzed using descriptive statistics and t-tests, while interview data were examined through thematic analysis.

**Results:**

The experimental group showed stronger improvement patterns in overall writing self-efficacy than the control group, including improvement in both task self-efficacy and skill self-efficacy. Within the experimental group, no statistically significant gender difference was observed in the subgroup comparisons, whereas differences were observed across internally defined pretest performance subgroups, with the higher pretest-performing subgroup showing a stronger improvement pattern than the lower pretest-performing subgroup. The interview findings provided supplementary insight into how students perceived the TSCA process and how these perceptions differed across subgroups.

**Conclusion:**

The findings suggest that, within the present instructional context, students in the TSCA group showed more favorable patterns of self-efficacy development than those in the comparison group. This pattern was observed in overall writing self-efficacy and in both subdimensions, although subgroup findings were exploratory and should be interpreted cautiously. Given the quasi-experimental design, the single-school sample, and the limited subgroup analyses, the results should be understood as context-bound rather than as strong causal evidence.

## 1 Introduction

Writing is a central component of second language (L2) learning and academic development, yet it is also one of the most demanding language skills for adolescent learners. Effective L2 writing requires not only linguistic knowledge but also higher-order processes such as planning, monitoring, revision, and emotional regulation. For secondary-level English as a foreign language (EFL) learners, these demands may be especially challenging because they are still developing both their language proficiency and their academic self-beliefs. In this context, learners’ writing self-efficacy (WSE), namely their beliefs about their capability to perform writing-related tasks successfully, has been recognized as an important factor influencing writing engagement, strategic effort, and performance (Bandura, 1997; Sun et al., 2021; Golparvar and Khafi, 2021). Grounded in social cognitive theory, self-efficacy is shaped by learners’ interpretations of their learning experiences, feedback, and perceived progress, and thus provides an important lens for understanding writing development in classroom settings.

Recent research has increasingly emphasized that assessment is not merely a mechanism for judging performance, but also a process that can influence students’ motivation, confidence, and engagement with learning. Within this perspective, feedback and assessment are considered most effective when students understand expectations, participate actively in evaluative processes, and use feedback to guide improvement (Winstone and Carless, 2020; Yu and Xu, 2022). For adolescent EFL learners, such processes may be particularly important because supportive and transparent assessment practices can help reduce uncertainty, strengthen learner agency, and foster more positive beliefs about writing. Prior studies have shown that students’ engagement with feedback is closely related to their affective responses and self-efficacy development (Dong et al., 2023; Yu et al., 2021). These findings suggest that assessment practices may play a meaningful role in shaping learners’ writing self-efficacy.

Against this background, collaborative and dialogic forms of assessment have attracted growing attention in L2 writing research. Rather than positioning students as passive recipients of teacher comments, collaborative assessment emphasizes interaction, negotiation, and shared evaluative judgment between teachers and learners. In L2 writing classrooms, teacher–student writing conferences and dialogic feedback practices have been shown to support revision, clarify writing goals, and strengthen students’ engagement with writing tasks (Yang, 2022; Goshu and Alemu, 2024). Related research on collaborative peer feedback has similarly indicated that co-constructed evaluative interactions can promote cognitive, affective, and behavioral engagement in writing (Chen et al., 2023; Peng et al., 2024). These developments make it theoretically plausible that teacher–student collaborative assessment (TSCA) may be associated with more favorable self-efficacy development by increasing transparency, participation, and perceived control during assessment.

However, several issues remain insufficiently explored in the existing literature. First, while collaborative assessment and feedback dialogue have received increasing attention in L2 writing research, a substantial proportion of published studies have been conducted in higher education settings, whereas secondary school learners have received comparatively less attention. Yet this population is especially relevant, since adolescent students are still forming academic self-beliefs and may be particularly sensitive to the emotional and motivational consequences of assessment. Second, although prior studies suggest that collaborative and dialogic assessment may support learner confidence, relatively limited research has directly examined the relationship between TSCA and L2 writing self-efficacy in secondary EFL contexts. Third, learner variables such as gender and proficiency have been discussed in the broader feedback and writing literature, but their possible relationship to TSCA-related self-efficacy development remains underexplored. In Chinese EFL classrooms, where writing assessment is still often teacher-dominated, these questions are especially worth investigating.

The present study therefore examines how teacher–student collaborative assessment was associated with Chinese senior high school students’ L2 writing self-efficacy in one secondary EFL context. More specifically, the study explores whether participation in TSCA was associated with changes in students’ writing self-efficacy, and whether such patterns differed across gender and internally defined pretest performance subgroups. By examining these questions in a secondary-level Chinese EFL context, this study seeks to contribute to a more nuanced understanding of how participatory assessment practices relate to students’ affective development in L2 writing classrooms.

To achieve this aim, the study addresses the following research questions:

RQ1. What patterns of change in L2 writing self-efficacy were observed among Chinese senior high school students under TSCA, compared with students receiving teacher-centered assessment?

RQ2. Do students of different genders show different patterns of L2 writing self-efficacy development under TSCA?

RQ3. Do students in different internally defined pretest performance subgroups show different patterns of L2 writing self-efficacy development under TSCA?

This study offers a context-specific contribution to the literature on collaborative assessment in L2 writing. By focusing on secondary-level Chinese EFL learners, it adds evidence from a comparatively less examined setting and explores how TSCA was associated with patterns of writing self-efficacy development in this context. The study also provides pedagogical reflections on more participatory and learner-supportive writing assessment practices in secondary EFL classrooms.

## 2 Related work

### 2.1 L2 writing development and writing self-efficacy

L2 writing is a cognitively and affectively demanding activity that requires the integration of linguistic resources, strategic processes, and psychological beliefs. Recent meta-analytic and empirical evidence suggests that writing self-efficacy (WSE) is one of the strongest predictors of L2 writing performance, strategy use, and cognitive regulation (Sun et al., 2021; Sun et al., 2021). Research also indicates that WSE influences students’ persistence, willingness to revise, and depth of engagement with teacher or peer feedback (Golparvar and Khafi, 2021; Fan, 2020; Fan and Xu, 2020).

Among adolescent EFL learners, WSE is closely tied to classroom affect, feedback experiences, and emotional responses to writing tasks. High-WSE learners demonstrate greater strategic flexibility and higher-order writing behaviors (Zhang et al., 2023; Li, 2022), whereas low-WSE learners tend to avoid challenging writing and engage less with feedback (Yu et al., 2021). Studies in integrated reading–writing tasks further show that componential WSE predicts both writing quality and source-based strategy deployment (Golparvar and Khafi, 2021), highlighting the need for pedagogical interventions that can strengthen adolescents’ writing confidence.

### 2.2 Formative and collaborative assessment

Formative assessment is increasingly conceptualized as a co-constructed, participatory process in which learners actively engage in meaning-making, evaluative judgment, and feedback uptake (Winstone and Carless, 2020; Dawson et al., 2023). Research on feedback literacy—students’ ability, dispositions, and actions to use feedback effectively—has expanded rapidly in recent years. Validated instruments for L2 writing feedback literacy and peer feedback literacy demonstrate that students who better understand criteria and quality standards engage more deeply and productively with feedback (Yu and Xu, 2022; Dong et al., 2023; Zhang et al., 2023).

At the same time, studies caution against the potential negative emotional impact of feedback, including threat-to-self and demotivation, especially in adolescent EFL populations (Yu et al., 2021; Turner, 2023). These findings underscore the need for feedback practices that support agency, reduce emotional costs, and promote constructive engagement. Research on dialogic feedback further reveals that teacher–student dialogue can facilitate disciplinary understanding, metacognitive reflection, and long-term writing development (Turner and Chan, 2021).

### 2.3 Teacher–student collaborative assessment (TSCA) in L2 writing

Teacher–student collaborative assessment (TSCA) foregrounds interaction, negotiation, and shared evaluative judgment. It differs from traditional teacher-driven assessment by positioning learners as active participants in analyzing writing quality and co-constructing revision plans. Studies of teacher–student writing conferences reveal improvements in accuracy, coherence, and engagement (Alfalagg and Alasmari, 2020; Yang, 2022). Recent evidence further shows that conferences help students articulate intentions, justify decisions, and build agency as writers (Goshu and Alemu, 2024).

Parallel work in collaborative peer feedback demonstrates that jointly produced feedback enhances affective, behavioral, cognitive, and social engagement (Chen et al., 2023; Peng et al., 2024). These findings help explain how TSCA may influence affective variables such as WSE: by making evaluation criteria visible, negotiable, and accessible.

Emerging classroom research with secondary EFL learners indicates that how students process teacher feedback—individually or through collaborative languaging—can lead to different learning outcomes, suggesting a mechanism through which TSCA may support confidence and deeper textual revision (Peng et al., 2024). Such findings imply that TSCA may work by providing structured, dialogic guidance that reduces uncertainty in writing tasks.

### 2.4 Research gaps and the present study

Despite promising evidence, several gaps remain. First, TSCA research in secondary-level EFL writing is scarce, with most studies conducted in tertiary contexts (Payant and Zuniga, 2022; Xhafaj, 2024). Second, while the concepts of evaluative judgment and feedback literacy offer theoretical explanations for how collaborative assessment may build learner confidence, their classroom translation—especially in teacher-centered systems—remains limited (Bearman et al., 2024; Sari et al., 2024).

Third, very few studies have examined whether patterns associated with TSCA in affective outcomes such as WSE vary by gender or learner subgroup, despite emerging evidence that feedback engagement and strategy use are influenced by individual differences (Weng et al., 2024; Turner, 2023). Finally, in the Chinese EFL context, writing assessment remains predominantly teacher-dominated, underscoring the need for research on participatory assessment approaches that may better enhance student engagement and writing confidence.

To address these gaps, the present study examines how TSCA was associated with Chinese senior high school EFL learners’ writing self-efficacy and further explores whether different patterns emerge across gender and internal pretest performance subgroups. By doing so, the study aims to enrich the growing body of collaborative assessment research and to offer pedagogical insight into more participatory and learner-supportive writing assessment practices in secondary EFL contexts.

## 3 Methods

### 3.1 Research design

This study adopted a quasi-experimental pretest–posttest design using two intact Grade 10 classes from the same public senior high school. One class was assigned as the experimental group and received teacher–student collaborative assessment (TSCA) in L2 writing instruction, while the other served as the control group and received conventional teacher-centered assessment.

A quasi-experimental design was adopted because random assignment of individual students was not feasible in the school context. The participating classes had already been administratively formed by the school before the study began, and reorganizing students across classes was not possible due to normal teaching arrangements and management constraints. Therefore, intact classes were used as the comparison units. Although this design allowed the intervention to be implemented under authentic classroom conditions, it also has limitations, including the possibility of selection bias and the reduced strength of causal inference compared with a fully randomized design.

To reduce major instructional differences between groups, both classes were taught by the same English teacher, followed the same syllabus, used the same textbook, and received the same amount of instructional time. At the same time, the dual role of the teacher as both instructor and researcher may have introduced expectancy-related bias. This issue was therefore considered when interpreting the findings, and the conclusions of the study were framed cautiously. Accordingly, the design was intended to support comparative analysis of observed change patterns across intact classes rather than strong causal inference about intervention effects.

This study employed a mixed-methods approach. Quantitative data were collected through a writing self-efficacy questionnaire and reading-into-writing tests administered before and after the intervention. Qualitative data were collected through semi-structured interviews with selected students from the experimental group in order to provide additional insight into how students perceived the TSCA process. Quantitative analyses were conducted using SPSS 29.0.

The intervention lasted 12 weeks, from 16 March 2024 to 28 June 2024, and was embedded in regular English writing instruction.

### 3.2 Participants

The participants were 90 Grade 10 students from two intact parallel classes at a key public senior high school in Shandong Province, China. Each class consisted of 45 students. One class was designated as the experimental group and received TSCA-based writing instruction, whereas the other served as the control group and continued with the school’s usual teacher-centered assessment approach.

In the experimental class, there were 20 boys and 25 girls; in the control class, there were 25 boys and 20 girls. Both classes were taught by the same English teacher using the same teaching materials, syllabus, and number of instructional hours, which helped reduce curricular and teacher-related variation across groups.

The participant selection was based on intact class availability in the target school and can therefore be characterized as school-based convenience sampling under authentic classroom conditions. Because the purpose of the study was to investigate the implementation of TSCA within a real secondary EFL setting, maintaining the natural class structure was considered pedagogically and administratively appropriate.

To explore possible subgroup differences, students in the experimental class were further divided into higher and lower pretest-performing subgroups on the basis of their writing pretest scores. Students in the upper 50% of the score distribution were assigned to the higher pretest-performing subgroup, while those in the lower 50% were assigned to the lower pretest-performing subgroup. This grouping procedure was intended only as an internal analytic distinction between relatively stronger and relatively weaker writers within the same instructional context. Because the grouping was based on internal pretest performance rather than on an external standardized proficiency measure, the subgroup findings should be interpreted cautiously and should not be treated as formal proficiency classifications.

Prior to data collection, permission to conduct the study was obtained from the school administration. Students and their legal guardians were informed of the purpose of the study and the procedures involved. Participation was voluntary, and participants were assured that all responses would remain anonymous and would be used solely for research purposes.

### 3.3 Instruments

#### 3.3.1 Reading-into-writing tests

Reading-into-writing tasks were administered as the pretest and posttest to assess students’ actual L2 writing performance and to examine baseline comparability between the two classes. The pretest task was taken from a Grade 10 provincial achievement test and consisted of a read-and-continue-writing task aligned with local curriculum requirements. Students first read a narrative passage and then completed the continuation in approximately 120–150 English words. The posttest used a task of comparable genre, length, and difficulty, also in a reading-into-writing format.

All compositions were scored independently by two experienced senior high school English teachers using the official National College Entrance Examination (Gaokao) English writing rubric adapted for reading-into-writing tasks. The rubric evaluated content development, organization, language accuracy, and vocabulary use. When the difference between the two raters exceeded 2 points, the script was re-examined and discussed until agreement was reached. The average of the two raters’ scores was then used for statistical analysis.

To provide evidence of scoring reliability, inter-rater reliability for the writing scores was estimated using the intraclass correlation coefficient (ICC). The result indicated satisfactory agreement between the two raters (ICC = 0.89). This double-rating procedure and reliability estimate were used to strengthen confidence in the writing scores, which were used both to examine baseline comparability and to construct the internal pretest performance subgroups in the experimental class.

#### 3.3.2 Writing self-efficacy questionnaire

Students’ L2 writing self-efficacy was measured using a 16-item Writing Self-Efficacy Scale adapted from existing Chinese EFL self-efficacy instruments reported in the literature and refined for the present study context. The adaptation involved minor wording adjustments so that the items aligned more closely with the writing tasks and classroom practices used in this study. The instrument consisted of two subdimensions:

1. Task self-efficacy (8 items): students’ confidence in completing different types of English writing tasks.
2. Skill self-efficacy (8 items): students’ confidence in using specific writing skills, such as organizing ideas, selecting vocabulary, applying grammar, and revising drafts.

Example items included “I am confident that I can complete an English writing task successfully” and “I am confident that I can organize my ideas clearly when writing in English.”

All items were rated on a five-point Likert scale ranging from 1 (strongly disagree) to 5 (strongly agree), with higher scores indicating stronger writing self-efficacy. The questionnaire was administered to both classes twice, once before the intervention and once after the 12-week intervention.

Before the main study, the adapted instrument was piloted with a comparable group of students. Reliability and basic construct-related evidence were examined using SPSS 29.0. Cronbach’s alpha coefficients were 0.909 for skill self-efficacy and 0.918 for task self-efficacy, indicating high internal consistency. The Kaiser–Meyer–Olkin (KMO) value was 0.917, and Bartlett’s test of sphericity was significant (*p* < 0.001), suggesting that the scale was suitable for factor-related examination and subsequent use in this study. Because the instrument was adapted for the present context, these results were used as preliminary evidence of appropriateness rather than as a full validation of the scale.

#### 3.3.3 Semi-structured interviews

To obtain qualitative insight into students’ experiences of TSCA, semi-structured interviews were conducted after the completion of the quantitative data collection. Twelve students were selected from the experimental class through purposive sampling so that different perspectives could be represented across gender and internal pretest performance subgroups. The selection was intended to support variation in interview perspectives rather than statistical representativeness. Where possible, the selection sought to include students from both gender groups and from relatively higher and lower pretest-performing subgroups within the experimental class.

The interview protocol focused on the following topics:

1. Perceived influence of TSCA on confidence in English writing
2. Perceived influence of TSCA on writing strategies and writing skills
3. Overall attitudes toward TSCA-based writing instruction
4. Possible influence of TSCA on other English learning experiences
5. Suggestions for improving TSCA in future classes.

Each interview lasted approximately 20–30 min and was conducted in Chinese so that students could express their ideas more freely and accurately. With participants’ consent, all interviews were audio-recorded and transcribed verbatim for analysis. The interview data were used analytically to help explain convergent and divergent patterns observed in the quantitative findings, rather than merely to provide illustrative comments.

### 3.4 Procedure

The 12-week intervention consisted of three major phases: pre-intervention preparation, instructional implementation, and post-intervention data collection.

#### 3.4.1 Pre-intervention phase

Before the intervention began, both classes completed the pretest reading-into-writing task and the pretest writing self-efficacy questionnaire in order to establish baseline information about students’ writing performance and writing self-efficacy. Students were informed that different forms of writing assessment would be used during the instructional period, but the specific research hypotheses were not disclosed so as to reduce expectancy effects.

#### 3.4.2 TSCA implementation in the experimental class

In the experimental class, TSCA was integrated into major writing lessons over the 12-week period. Each instructional cycle followed a three-stage procedure:

1. Pre-class preparation: Students completed a writing task as assigned work. The teacher read all drafts, identified common strengths and weaknesses, and selected representative samples for classroom discussion. Based on the drafts, the teacher determined the main assessment focus for the next lesson, such as coherence, tense consistency, idea development, or argument support.
2. In-class collaborative assessment: At the beginning of the lesson, the teacher and students revisited the scoring criteria together. Students then participated in peer-assessment and self-assessment activities in small groups using checklist-based prompts derived from the rubric. After this, the teacher guided whole-class discussion of selected sample scripts, inviting students to explain their judgments, compare evaluations, and discuss revision possibilities. During this process, the teacher provided clarification, scaffolding, and formative feedback, while encouraging students to ask questions and reflect on their own writing.
3. Post-class revision and consolidation: Students revised their compositions based on the collaborative feedback and submitted a second draft. The teacher then reviewed the revised drafts and provided additional comments when necessary. In some lessons, an online composition platform was used to store drafts and feedback so that students could review earlier comments and monitor their progress.

This cycle was implemented six times across the 12-week intervention period as part of regular writing instruction. To help maintain procedural consistency, the same teacher followed the same three-stage TSCA procedure throughout the intervention. Although formal implementation fidelity was not independently measured, lesson planning and classroom implementation were guided by a consistent instructional routine across the six instructional cycles. The purpose of the TSCA procedure was to transform assessment from a one-way teacher judgment process into a dialogic, participatory, and reflective activity in which students actively engaged with criteria, evaluation, and revision.

#### 3.4.3 Traditional teacher-centered assessment in the control class

In the control class, writing instruction followed the school’s usual teacher-centered assessment pattern. Students completed writing assignments individually and submitted them to the teacher. The teacher corrected all drafts, summarized common problems, and selected several model compositions for classroom explanation. In class, students mainly listened to the teacher’s feedback and took notes, and after class they revised their drafts individually before resubmission.

Although students could read teacher comments and occasionally ask questions, they were not systematically involved in co-constructing criteria, evaluating sample scripts, or participating in collaborative judgment activities. This condition therefore represented the conventional teacher-led assessment approach against which TSCA was compared.

#### 3.4.4 Post-intervention data collection

At the end of the 12-week intervention, both classes completed the posttest reading-into-writing task and the posttest writing self-efficacy questionnaire under the same conditions as the pretest. After the quantitative phase was completed, semi-structured interviews were conducted with selected students from the experimental class.

### 3.5 Data analysis

Quantitative data from the questionnaire and writing tests were entered into Excel and then analyzed in SPSS 29.0. The main analytical procedures included:

1. Reliability and preliminary validity-related analysis of the self-efficacy scale, including Cronbach’s alpha, KMO, and Bartlett’s test.
2. Descriptive statistics, including means and standard deviations, for writing scores and self-efficacy scores at pretest and posttest.
3. Independent-samples t-tests to examine baseline equivalence between the experimental and control groups and to compare posttest performance across groups.
4. Paired-samples t-tests to examine pre–post changes within groups and, where relevant, within subgroups.
5. Additional subgroup comparisons to explore patterns associated with gender and internal pretest performance subgroup membership.

Before conducting the t-tests, the assumptions of normality and homogeneity of variance were examined. Normality was checked using the Shapiro–Wilk test, and homogeneity of variance for independent-samples t-tests was assessed using Levene’s test. The results indicated that the assumptions were sufficiently met for the present analyses.

Given the relatively small sample size, the intact-class design, and the exploratory nature of the subgroup analyses, the quantitative analyses were primarily intended to describe within-sample change patterns and between-group differences in a transparent and interpretable way. Accordingly, descriptive statistics and t-tests were used as the main analytic procedures, while subgroup findings were treated as exploratory rather than as evidence of formal moderation. Given the quasi-experimental design and intact-class structure, the statistical analyses were intended to identify group differences and change patterns within the current sample rather than to support overly strong causal claims. Therefore, findings related to subgroup patterns were interpreted cautiously.

Qualitative interview data were analyzed using thematic analysis following Braun and Clarke (2006). After transcription, the interview texts were read repeatedly to develop familiarity with the data. Initial inductive codes were then generated from participants’ responses, after which related codes were grouped into broader themes concerning confidence, writing strategies, participation in assessment, and perceptions of TSCA. The themes were reviewed and refined through repeated comparison with the original transcripts to ensure interpretive consistency. To strengthen trustworthiness, a coding record was maintained throughout the analytic process, and the developing thematic structure was discussed and reviewed with the second author. In the presentation of qualitative findings, interview evidence was used primarily to complement and help interpret the quantitative results rather than to make independent generalizable claims.

### 3.6 Ethics statement

This study involved human participants in the form of classroom-based questionnaire administration, writing tasks, and interviews. The research was conducted within regular instructional practice and involved minimal risk. Permission to conduct the study was obtained from the school administration. Prior to data collection, students and their legal guardians were informed of the purpose of the study, and informed consent was obtained. Participation was voluntary, and all collected data were anonymized and used solely for research purposes.

## 4 Results

### 4.1 Patterns of L2 writing self-efficacy development associated with TSCA

This section reports the quantitative results concerning patterns of L2 writing self-efficacy development associated with teacher–student collaborative assessment (TSCA). To examine whether the experimental and control groups were comparable before the intervention, pretest data were first analyzed. Posttest comparisons were then conducted to evaluate group differences after the 12-week intervention. Finally, within-group pre–post comparisons were used to describe changes in writing self-efficacy over time. Preliminary analyses showed no serious violations of the assumptions underlying the t-tests used in this study.

#### 4.1.1 Pretest comparison of writing self-efficacy

Before the intervention, both the experimental class and the control class completed the Writing Self-Efficacy Questionnaire. A total of 90 valid questionnaires were collected. Descriptive statistics for the pretest are presented in Table 1, and the independent-samples t-test results are shown in Table 2.

**Table 1:** Descriptive statistics for pretest questionnaire.

Variable	Group	*N*	Mean	SD	SE Mean
Task writing Self-efficacy	Experimental class	45	3.269	0.709	0.105
	Control class	45	3.230	0.710	0.105
Skill writing Self-efficacy	Experimental class	45	3.433	0.820	0.122
	Control class	45	3.297	0.773	0.115
Overall writing Self-efficacy	Experimental class	45	3.375	0.724	0.107
	Control class	45	3.351	0.695	0.103

**Table 2:** Independent-samples t-test results for pretest writing self-efficacy by group.

Variable	Experimental M (SD)	Control M (SD)	t	df	*p*	Cohen’s d
Task writing self-efficacy	3.269 (0.709)	3.230 (0.710)	−0.260	88	0.796	0.05
Skill writing self-efficacy	3.433 (0.820)	3.297 (0.773)	−0.810	88	0.420	0.17
Overall writing self-efficacy	3.375 (0.724)	3.351 (0.695)	−0.585	88	0.560	0.03

As shown in Table 1, the experimental and control groups displayed similar mean scores in task self-efficacy, skill self-efficacy, and overall writing self-efficacy at the pretest stage. The corresponding standard deviations also suggested comparable score distributions across the two groups.

As shown in Table 2, no statistically significant between-group differences were observed at pretest in overall writing self-efficacy or in either subdimension, suggesting that the two intact classes were broadly comparable at baseline.

#### 4.1.2 Posttest comparison of writing self-efficacy

After the 12-week instructional intervention, both groups completed the posttest Writing Self-Efficacy Questionnaire. Descriptive statistics are reported in Table 3, and the independent-samples t-test results are reported in Table 4.

**Table 3:** Descriptive statistics for posttest questionnaire.

Variable	Group	*N*	Mean	SD	SE Mean
Task writing Self-efficacy	Experimental class	45	3.775	0.632	0.094
	Control class	45	3.436	0.498	0.074
Skill writing Self-efficacy	Experimental class	45	3.977	0.561	0.083
	Control class	45	3.561	0.594	0.088
Overall writing Self-efficacy	Experimental class	45	3.876	0.563	0.074
	Control class	45	3.498	0.497	0.083

**Table 4:** Independent-samples t-test results for posttest writing self-efficacy by group.

Variable	Experimental M (SD)	Control M (SD)	t	df	*p*	Cohen’s d
Task writing self-efficacy	3.775 (0.632)	3.436 (0.498)	−2.824	88	0.006	0.60
Skill writing self-efficacy	3.977 (0.561)	3.561 (0.594)	−3.417	88	< 0.001	0.72
Overall writing self-efficacy	3.876 (0.563)	3.498 (0.497)	−3.373	88	0.001	0.71

As shown in Table 3, the experimental group obtained higher posttest mean scores than the control group in task self-efficacy, skill self-efficacy, and overall writing self-efficacy. This pattern suggests that students receiving TSCA reported stronger writing self-efficacy after the intervention period than those receiving traditional teacher-centered assessment.

As shown in Table 4, statistically significant between-group differences were observed at posttest in overall writing self-efficacy as well as in both subdimensions. These results suggest that, within the present sample, the experimental group showed a more favorable posttest self-efficacy profile than the control group.

#### 4.1.3 Within-group pre–post comparisons

To further describe how writing self-efficacy changed over time within each group, paired-samples t-tests were conducted separately for the control class and the experimental class. The results are presented in Tables 5, 6.

**Table 5:** Paired-samples t-test results for pretest–posttest changes in writing self-efficacy in the control group.

Variable	Pretest M (SD)	Posttest M (SD)	Mean difference	t	df	*p*	Cohen’s dz
Task writing self-efficacy	3.230 (0.710)	3.436 (0.498)	0.206	1.848	44	0.071	0.28
Skill writing self-efficacy	3.297 (0.773)	3.561 (0.594)	0.264	0.497	44	0.622	0.07
Overall writing self-efficacy	3.351 (0.695)	3.498 (0.497)	0.147	0.660	44	0.513	0.10

**Table 6:** Paired-samples t-test results for pretest–posttest changes in writing self-efficacy in the experimental group.

Variable	Pretest M (SD)	Posttest M (SD)	Mean difference	t	df	*p*	Cohen’s dz
Task writing self-efficacy	3.269 (0.709)	3.775 (0.632)	0.506	2.858	44	0.006	0.43
Skill writing self-efficacy	3.433 (0.820)	3.977 (0.561)	0.544	2.662	44	0.011	0.40
Overall writing self-efficacy	3.375 (0.724)	3.876 (0.563)	0.501	2.066	44	0.045	0.31

In the control group, mean scores increased slightly from pretest to posttest in overall writing self-efficacy and in both subdimensions. However, the overall pattern of change remained relatively modest, as shown in Table 5.

In the experimental group, mean scores increased from pretest to posttest in overall writing self-efficacy as well as in task and skill self-efficacy. As shown in Table 6, this group displayed a clearer within-group improvement pattern over the intervention period.

#### 4.1.4 Summary of quantitative findings

Taken together, the between-group posttest comparisons and the within-group pre–post results suggest that, within the present sample, the TSCA group showed a more favorable pattern of change in L2 writing self-efficacy than the comparison group. In particular, the experimental group showed stronger posttest scores and clearer within-group improvement in overall writing self-efficacy and in both subdimensions.

At the same time, because this study employed a quasi-experimental design with intact classes rather than random assignment, these findings should be interpreted as evidence of an association between TSCA and more favorable self-efficacy development within the current instructional context, rather than as definitive proof of causal effect.

### 4.2 Gender-related patterns in writing self-efficacy within the experimental group

This section explores whether boys and girls in the experimental group displayed different patterns of writing self-efficacy before and after the TSCA intervention. The analysis is based on subgroup comparisons within the experimental class and is intended to provide a descriptive view of gender-related patterns rather than a formal test of interaction.

#### 4.2.1 Quantitative comparison between boys and girls

Descriptive statistics for boys and girls in the experimental class at pretest and posttest are presented in Table 7. The independent-samples t-test results are reported in Tables 8, 9.

**Table 7:** Descriptive statistics of pretest and posttest for boys and girls.

Group	N	Mean	SD	SE Mean
Male (pretest)	20	3.017	0.859	0.189
Female (pretest)	25	3.065	0.586	0.117
Male (posttest)	20	3.578	0.528	0.184
Female (posttest)	25	3.677	0.868	0.147

**Table 8:** Independent-samples t-test results for pretest gender comparison within the experimental group.

Comparison	Male M (SD)	Female M (SD)	t	df	*p*	Cohen’s d
Pretest writing self-efficacy	3.017 (0.859)	3.065 (0.586)	0.583	42	0.563	0.07

**Table 9:** Independent-samples t-test results for posttest gender comparison within the experimental group.

Comparison	Male M (SD)	Female M (SD)	t	df	*p*	Cohen’s d
Posttest writing self-efficacy	3.578 (0.528)	3.677 (0.868)	−1.25	42	0.217	0.14

As shown in Table 7, the mean writing self-efficacy scores of boys and girls were relatively close at pretest. After the intervention, both groups showed higher mean scores than before, indicating that both boys and girls reported improvement in writing self-efficacy following participation in TSCA.

As shown in Table 8, no statistically significant pretest difference was observed between boys and girls in the experimental class. The mean scores of the two gender groups were relatively close at baseline, suggesting that they entered the intervention with broadly similar levels of writing self-efficacy.

As shown in Table 9, no statistically significant posttest difference was observed between boys and girls within the experimental class. Descriptively, both groups showed higher mean scores after the intervention, and the overall pattern suggests broadly similar self-efficacy development across gender groups. Given the limited sample size and the absence of a formal interaction analysis, these subgroup results should be interpreted cautiously.

#### 4.2.2 Qualitative insights into gender-related patterns

To complement the subgroup comparisons, interview data were examined to explore how male and female students in the experimental class perceived the TSCA process. Overall, students from both groups described TSCA as a more transparent, participatory, and supportive assessment experience than conventional teacher-centered evaluation.

Across interviews, both male and female students reported that TSCA helped them better understand writing criteria and become more aware of how their writing could be improved. Several students noted that classroom discussion and teacher–student dialogue made assessment standards clearer and gave them more confidence when revising their drafts. Students from both groups also emphasized that participating in evaluation, rather than only receiving teacher comments, increased their sense of involvement in the writing process.

At the same time, the interview responses did not reveal a clearly contrasting pattern between boys and girls. Although individual students expressed themselves in slightly different ways, the overall themes were broadly similar across gender groups. Both groups referred to clearer expectations, greater engagement in revision, and stronger confidence after participating in collaborative assessment activities.

These qualitative findings are broadly consistent with the quantitative subgroup comparisons, which did not show a statistically significant gender difference within the experimental class. However, the interview data should be interpreted as supplementary evidence for understanding students’ perceptions rather than as definitive proof that gender played no role in self-efficacy development. A more rigorous examination of gender-related effects would require a formal interaction analysis in future research.

### 4.3 Patterns in writing self-efficacy across internal pretest performance subgroups within the experimental group

This section examines whether students in different internal pretest performance subgroups within the experimental group displayed different patterns of writing self-efficacy development during the TSCA intervention. As in the gender analysis, the results should be interpreted as subgroup comparison findings rather than as a formal moderation test.

#### 4.3.1 Quantitative comparison between higher and lower pretest-performing subgroups

Before the intervention, students in the experimental class were divided into higher and lower pretest-performing subgroups based on their writing pretest scores. Descriptive statistics are presented in Table 10, and the independent-samples t-test results are shown in Tables 11, 12.

**Table 10:** Descriptive statistics for writing self-efficacy across internal pretest performance subgroups within the experimental group.

Group	*N*	Mean	SD	SE Mean
Higher pretest-performing subgroup (pretest)	22	3.142	0.674	0.159
Lower pretest-performing subgroup (pretest)	23	3.029	0.498	0.145
Higher pretest-performing subgroup (posttest)	22	3.679	0.458	0.097
Lower pretest-performing subgroup (posttest)	23	3.203	0.755	0.157

**Table 11:** Independent-samples t-test results for pretest comparison across internal pretest performance subgroups within the experimental group.

Comparison	Higher pretest-performing subgroup M (SD)	Lower pretest-performing subgroup M (SD)	t	df	*p*	Cohen’s d
Pretest writing self-efficacy	3.142 (0.674)	3.029 (0.498)	−0.921	43	0.363	0.19

**Table 12:** Independent-samples t-test results for posttest comparison across internal pretest performance subgroups within the experimental group.

Comparison	Higher pretest-performing subgroup M (SD)	Lower pretest-performing subgroup M (SD)	t	df	*p*	Cohen’s d
Posttest writing self-efficacy	3.679 (0.458)	3.203 (0.755)	−3.088	43	0.004	0.76

As shown in Table 10, the higher and lower pretest-performing subgroups had relatively similar writing self-efficacy mean scores at pretest. As shown in Table 11, no statistically significant pretest difference was observed between the two subgroups, suggesting that their initial writing self-efficacy levels were broadly comparable within the experimental class.

After the TSCA intervention, both subgroups showed higher self-efficacy scores than before, but the increase was descriptively larger for the higher pretest-performing subgroup. As shown in Table 12, a statistically significant posttest difference was observed between the two internal pretest performance subgroups within the experimental class.

These results suggest that differences emerged across the internal pretest performance subgroups in writing self-efficacy development during the intervention period, with the higher pretest-performing subgroup showing a stronger improvement pattern in the current sample. However, because the subgroup analysis was based on internal grouping within one experimental class and did not involve a formal moderation model, the results should be interpreted cautiously.

#### 4.3.2 Qualitative insights into patterns across internal pretest performance subgroups

Interview data were also used to further examine how students in different internal pretest performance subgroups experienced TSCA. In general, both higher and lower pretest-performing students described the collaborative assessment process as helpful, but the nature of their reported benefits appeared somewhat different.

Higher pretest-performing students tended to report that TSCA helped them clarify writing standards, refine revision decisions, and engage more actively in reflective discussion. They generally described themselves as being able to understand teacher feedback more readily and to apply suggestions more effectively in subsequent revisions. For these students, collaborative assessment appeared to provide an opportunity to strengthen confidence through more active participation and more precise control over writing improvement.

Lower pretest-performing students also expressed positive attitudes toward TSCA, particularly in relation to receiving support and clearer guidance. However, some of them reported that they still found it difficult to interpret certain feedback comments fully or to translate evaluative suggestions into effective revisions. Their responses suggest that although TSCA created a more supportive environment, students with weaker initial writing foundations may have needed more explicit scaffolding in order to benefit from the process to the same extent as their higher pretest-performing peers.

These qualitative observations are broadly consistent with the quantitative subgroup comparisons, which showed a stronger improvement pattern for the higher pretest-performing subgroup. At the same time, the interview evidence should not be interpreted as conclusive proof of statistical moderation. Rather, it offers a possible explanatory perspective on why differences across internal pretest performance subgroups emerged in the present sample. Future studies with larger samples and more robust analytical designs are needed to examine these patterns more rigorously.

## 5 Discussion

### 5.1 Summary of main findings

The present study examined the relationship between teacher–student collaborative assessment (TSCA) and senior high school students’ L2 writing self-efficacy in a Chinese EFL context. Based on the quantitative and qualitative results, three main findings emerged.

First, students in the experimental class who participated in TSCA showed a more favorable pattern of improvement in overall writing self-efficacy than those in the comparison class who experienced conventional teacher-centered assessment. This pattern was reflected not only in overall writing self-efficacy but also in the two subdimensions of task self-efficacy and skill self-efficacy. Within the current sample, the findings suggest that TSCA was associated with more favorable self-efficacy development during the intervention period.

Second, the subgroup comparisons within the experimental class did not reveal a statistically significant difference between boys and girls in writing self-efficacy at either pretest or posttest. In descriptive terms, both groups showed improvement after the intervention, and the interview data also suggested broadly similar perceptions of the TSCA process. However, because a formal interaction analysis was not conducted, these results should be interpreted as indicating similar subgroup patterns rather than as definitive evidence regarding the role of gender in TSCA-related self-efficacy development.

Third, differences across internal pretest performance subgroups were observed within the experimental class. Although both higher and lower pretest-performing subgroups showed improvement, the higher pretest-performing subgroup displayed a stronger improvement pattern in the present sample. The interview findings offered possible explanatory insight into this pattern, suggesting that students with relatively stronger initial writing performance may have found it easier to interpret criteria and apply feedback during collaborative assessment activities. At the same time, the present analyses do not justify strong statistical claims of moderation, and the subgroup findings should therefore be interpreted with caution.

Taken together, the results suggest that TSCA may provide a useful assessment context for supporting adolescent learners’ confidence in L2 writing, while the extent of observed benefit may vary across learners with different levels of initial writing performance within the same class.

### 5.2 Interpreting the relationship between TSCA and writing self-efficacy

The more favorable improvement pattern observed in the experimental group may be interpreted in light of Bandura’s social cognitive theory, particularly the role of self-efficacy in shaping learners’ motivation, persistence, and performance-related beliefs. From this perspective, learners’ self-efficacy is influenced by how they interpret task demands, feedback experiences, and their own progress. In the present study, TSCA may have supported students’ self-efficacy by making assessment criteria more visible, increasing students’ participation in evaluative processes, and allowing them to engage more actively with revision.

Compared with conventional teacher-centered assessment, TSCA provided more opportunities for students to discuss evaluation standards, reflect on their own writing, and receive feedback in a dialogic form. Such experiences may have helped students feel that writing tasks were more manageable and that improvement was more attainable. In Bandura’s terms, these classroom experiences may have contributed to stronger efficacy beliefs by providing students with more structured opportunities to interpret performance-related information in constructive ways.

This interpretation is also consistent with previous research suggesting that student engagement with feedback and evaluative processes can strengthen confidence and strategic involvement in writing (e.g., Winstone and Carless, 2020; Yu and Xu, 2022). In the present study, TSCA appeared to function not simply as an assessment technique, but as a classroom process through which students could better understand writing expectations and become more engaged participants in their own improvement.

At the same time, it is important not to overstate the findings. Because the study employed a quasi-experimental design with intact classes and was conducted in a single school, the results should not be interpreted as definitive causal proof that TSCA directly increases writing self-efficacy. Rather, the findings suggest that TSCA was associated with a more favorable pattern of self-efficacy development within the present instructional context.

### 5.3 Gender-related patterns

The quantitative subgroup comparisons did not reveal a statistically significant difference between boys and girls in writing self-efficacy within the experimental class at either pretest or posttest. In addition, interview responses suggested that students from both gender groups generally perceived TSCA as a clearer, more participatory, and more supportive assessment experience.

One possible interpretation is that the core features of TSCA, including shared criteria, dialogue, and active participation in evaluation, may provide similar opportunities for engagement across gender groups. When students are given comparable access to task expectations, revision guidance, and evaluative discussion, differences in perceived participation may become less pronounced. In this sense, the collaborative structure of TSCA may contribute to a more balanced classroom environment.

However, the present findings should not be interpreted too strongly. The study did not conduct a formal gender-by-treatment interaction analysis, and the subgroup comparisons were based on a relatively limited sample. Therefore, it would be more accurate to conclude that no statistically significant gender difference was observed in the present subgroup comparisons, rather than to draw broader conclusions about the role of gender in TSCA-related self-efficacy development.

### 5.4 Patterns across internal pretest performance subgroups

Unlike the gender comparisons, the subgroup analyses based on internal pretest writing performance suggested that students in the higher pretest-performing subgroup showed a stronger improvement pattern in self-efficacy during the TSCA intervention. A possible explanation is that students with relatively stronger initial writing performance may have had greater capacity to understand evaluative criteria, interpret teacher feedback, and translate collaborative discussion into concrete revision strategies.

From a social cognitive perspective, learners with stronger prior linguistic and strategic resources may also be more likely to interpret collaborative assessment experiences as evidence of growing competence. In contrast, students with relatively weaker initial writing performance may require more time, more explicit guidance, and more structured support before similar assessment experiences can translate into stronger self-efficacy beliefs.

The interview data offered a broadly consistent picture. Students in the higher pretest-performing subgroup more often described TSCA as helping them refine ideas, understand criteria, and engage more confidently in revision, whereas students in the lower pretest-performing subgroup tended to emphasize the supportive value of the process while also reporting difficulty in using feedback independently. This pattern suggests that collaborative assessment may not be experienced in the same way by all learners, even within the same classroom setting.

Nevertheless, these interpretations should remain cautious. The subgrouping was based on internal pretest performance within one experimental class rather than on an external standardized proficiency measure, and no formal moderation model was tested. Therefore, the present study supports the interpretation that differences emerged across internally defined pretest performance subgroups in the sample, rather than the stronger claim that proficiency statistically moderated the role of TSCA.

### 5.5 Pedagogical implications

Despite the limitations noted above, the findings offer several pedagogical implications for secondary EFL writing instruction.

First, the study suggests that collaborative and dialogic assessment practices may be beneficial for supporting students’ confidence in writing. By involving students more actively in discussing criteria, interpreting feedback, and revising drafts, TSCA may help create a more participatory assessment environment than conventional teacher-centered approaches.

Second, the findings highlight the importance of transparency in writing assessment. When students understand the basis on which writing is evaluated and are encouraged to engage in evaluative dialogue, they may be better positioned to develop stronger confidence and more active revision behaviors.

Third, the results indicate that differentiated support may be especially important for students with relatively weaker initial writing performance. Although these students may still benefit from collaborative assessment, they may require more explicit scaffolding, modeling, simplified checklists, or guided practice in order to engage fully with evaluative dialogue and make productive use of feedback.

Finally, the study underscores that classroom assessment should be considered not only as a means of judging performance but also as a process that can shape students’ beliefs about themselves as writers. In this sense, assessment design may play an important role in creating conditions that support learner engagement and confidence.

### 5.6 Limitations of the study

Several limitations should be acknowledged.

First, the study was conducted with two intact classes from a single senior high school, which limits the generalizability of the findings. Second, the intervention lasted only 12 weeks, and therefore longer-term developments in writing self-efficacy remain unknown. Third, the study relied heavily on self-report questionnaire data, which may be influenced by subjective perception. Fourth, the subgroup analyses related to gender and internal pretest performance were based on relatively limited sample sizes and simple comparison procedures; therefore, these findings should be interpreted cautiously. Fifth, while the interview data provided useful supplementary insight, the qualitative component was intended mainly to support interpretation of the quantitative findings rather than to function as a fully independent qualitative investigation.

In addition, because the study used a quasi-experimental design with intact classes and the teacher also served as the researcher, causal interpretations should remain conservative. Although several steps were taken to reduce instructional variation across groups, potential bias cannot be completely ruled out.

### 5.7 Directions for future research

Future research could extend the present study in several ways. First, studies involving larger and more diverse school samples are needed to examine whether similar patterns emerge across different secondary EFL contexts. Second, longer-term research would be valuable for investigating whether the observed self-efficacy development is sustained over time. Third, future studies could adopt more robust analytical designs, such as ANCOVA, repeated-measures models, or mixed-effects models, to examine intervention effects and subgroup differences more rigorously.

In addition, further research could explore more detailed class-wise or learner-level processes, especially among students with relatively weaker initial writing performance, who may need additional scaffolding in collaborative assessment settings. Finally, future studies may investigate how TSCA relates not only to self-efficacy but also to other affective and behavioral variables, such as motivation, engagement, and anxiety, which were not directly measured in the present study.

## 6 Conclusion

The present study investigated how teacher–student collaborative assessment (TSCA) was related to senior high school students’ L2 writing self-efficacy in a Chinese EFL context. Drawing on quantitative questionnaire data and supplementary interview evidence, the study identified several noteworthy patterns.

First, students in the experimental class showed a more favorable pattern of improvement in overall writing self-efficacy than those in the control class over the 12-week intervention period. This pattern was also reflected in task self-efficacy and skill self-efficacy. Within the present instructional context, these findings suggest that TSCA was associated with more favorable development of students’ confidence in L2 writing than conventional teacher-centered assessment.

Second, the subgroup comparisons did not reveal a statistically significant gender difference within the experimental class. Both boys and girls showed positive development in writing self-efficacy, and the interview data suggested broadly similar perceptions of the TSCA process. However, because no formal interaction analysis was conducted, this finding should be interpreted cautiously.

Third, differences across internal pretest performance subgroups were observed in the experimental group. Students in the higher pretest-performing subgroup showed a stronger improvement pattern than those in the lower pretest-performing subgroup. The qualitative data suggested that students with relatively stronger initial writing performance may have found it easier to interpret criteria and make use of collaborative feedback, whereas students with relatively weaker initial writing performance may have required more explicit support. These findings point to the importance of differentiated scaffolding in collaborative assessment contexts.

Overall, the study suggests that TSCA was associated with a more favorable pattern of writing self-efficacy development in this secondary EFL context. The observed pattern may reflect the role of dialogue, shared evaluative judgment, and more active student participation in assessment, although such interpretation should remain cautious. At the same time, the findings should be understood in light of the study’s limitations, including the quasi-experimental design, the single-school sample, and the relatively limited subgroup analyses.

Future research is needed to examine the long-term effects of TSCA, to test subgroup differences using more robust statistical models, and to investigate how collaborative assessment interacts with other affective and cognitive dimensions of L2 writing development.

## Data Availability

The raw data supporting the conclusions of this article will be made available by the authors, without undue reservation.
